# Supplementary material for: Using Drosophila behavioral assays to characterize terebrid venom-peptide bioactivity
Source: Sci Rep. 2018 Oct 15;8:15276. doi: 10.1038/s41598-018-33215-2 (PMC6189199; doi:10.1038/s41598-018-33215-2)
Supplement: Supplementary file 1 — Supplementary Information [file 41598_2018_33215_MOESM1_ESM.docx]

Supplementary document

Using *Drosophila* behavioral assays to characterize terebrid venom-peptide bioactivity

Anders Eriksson^1+^, Prachi Anand^2,3+^, Juliette Gorson^2,3,4,5^, Corina Grijuc^2^, Elina Hadelia^2^, James C Stewart^1^, Mandë Holford ^2,3,4,5,6*^, Adam Claridge-Chang ^1,7,8,*^

^1^Institute of Molecular and Cell Biology, 61 Biopolis Drive, Singapore 138673

^2^Department of Chemistry, Hunter College Belfer Research Center, New York, NY 10021

^3^Department of Biochemistry, Weill Cornell Medical College, Cornell University, New York, NY 10021

^4^Division of Invertebrate Zoology, The American Museum of Natural History, New York, NY 10024

^5^Program in Biology, The Graduate Center, City University of New York, New York, NY 10016

^6^Program in Chemistry & Biochemistry, The Graduate Center, City University of New York, New York, NY 10016

^7^Duke-NUS Medical School, 61 Biopolis Drive, Singapore 138673

^8^Department of Physiology, NUS Yong Loo Lin School of Medicine, Singapore 138673

^+^ Co-first authors

*Correspondence: [claridge-chang.adam@duke-nus.edu.sg](mailto:claridge-chang.adam@duke-nus.edu.sg); [mholford@hunter.cuny.edu](mailto:mholford@hunter.cuny.edu)


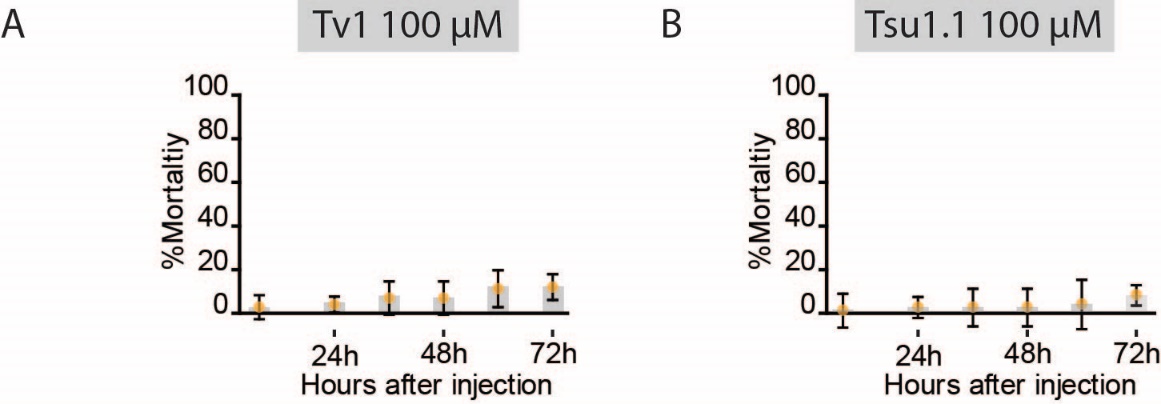


**Figure S1. Mortality effects of Tv1 and Tsu1.1** The venom peptides Tv1 and Tsu1.1 have little if any effect on 72-h mortality. The percent mortality is corrected for the mortality of vehicle-treated controls.

2.30

**Figure S2. Solid-phase synthesis of Tsu1.1 peptide.** RP-UHPLC chromatogram of Tsu1.1 linear peptide at 214nm. Linear peptide show a single peak at 2.3 min on a gradient of 0% to 35% B (80% acetonitrile in water).

**Figure S3. Mass Spectrometry analyses of linear Ts1.1** LC-MS showing the monoisotopic mass of the linear peptide 2194.92Da (M+1) with the expected mass of 2194.88 (M+1). Spectrum shows m/z of +2 as 1098.46 and +3 as 732.65Da.

1.52

**Figure S4. Oxidative folding of Tsu1.1** RP-UHPLC chromatogram of Tsu1.1 fully oxidized peptide at 214nm. Folded peptide show a single peak at 1.52 min on a gradient of 0% to 35% B (80% acetonitrile in water).

**Figure S5: Mass Spectrometry analyses of folded Ts1.1**LC-MS showing the monoisotopic mass of the fully oxidized peptide as 2190.86Da with the expected mass of 2190.88. Spectrum shows m/z of +2 as 1096.43, +3 as 731.28 and +4 as 548.72Da.
